# The early Cambrian fossil embryo *Pseudooides* is a direct-developing cnidarian, not an early ecdysozoan

**DOI:** 10.1098/rspb.2017.2188

**Published:** 2017-12-13

**Authors:** Baichuan Duan, Xi-Ping Dong, Luis Porras, Kelly Vargas, John A. Cunningham, Philip C. J. Donoghue

**Affiliations:** 1 Research Center for Islands and Coastal Zone, First Institute of Oceanography, State Oceanic Administration, Qingdao 266061, People's Republic of China; 2 School of Earth and Space Science, Peking University, Beijing 100871, People's Republic of China; 3 School of Earth Sciences, University of Bristol, Life Sciences Building, Tyndall Avenue, Bristol BS8 1TQ, UK

**Keywords:** *Pseudooides*, embryo, development, Cambrian, Cnidaria, Ecdysozoa

## Abstract

Early Cambrian *Pseudooides prima* has been described from embryonic and post-embryonic stages of development, exhibiting long germ-band development. There has been some debate about the pattern of segmentation, but this interpretation, as among the earliest records of ecdysozoans, has been generally accepted. Here, we show that the ‘germ band’ of *P. prima* embryos separates along its mid axis during development, with the transverse furrows between the ‘somites’ unfolding into the polar aperture of the ten-sided theca of *Hexaconularia sichuanensis*, conventionally interpreted as a scyphozoan cnidarian; co-occurring post-embryonic remains of ecdysozoans are unrelated. We recognize *H. sichuanensis* as a junior synonym of *P. prima* as a consequence of identifying these two form-taxa as distinct developmental stages of the same organism. Direct development in *P. prima* parallels the co-occuring olivooids *Olivooides,* and *Quadrapyrgites* and Bayesian phylogenetic analysis of a novel phenotype dataset indicates that, despite differences in their tetra-, penta- and pseudo-hexa-radial symmetry, these hexangulaconulariids comprise a clade of scyphozoan medusozoans, with *Arthrochites* and conulariids, that all exhibit direct development from embryo to thecate polyp. The affinity of hexangulaconulariids and olivooids to extant scyphozoan medusozoans indicates that the prevalence of tetraradial symmetry and indirect development are a vestige of a broader spectrum of body-plan symmetries and developmental modes that was manifest in their early Phanerozoic counterparts.

## 1. Introduction

Embryos are among the earliest unequivocal animal fossil remains in the geological record and, as such, they have the potential to provide fundamental insights into the embryology of animals during the evolutionary emergence of the fundamental metazoan body plans. Unfortunately, the phylogenetic affinity and, thus, the evolutionary significance of most remain unresolved [1]. This is especially true of *Pseudooides prima*, originally described from spherical fossils of unknown affinity from the earliest Cambrian Kuanchuanpu Formation and equivalents from southern Shaanxi Province and northern Sichuan Province, South China [2,3]. *Pseudooides* fossils were subsequently interpreted as ecdysozoan embryos based on evidence of irregular spiral/radial cleavage, pinched germ bands comprising six paired, bilaterally arranged domains, interpreted to exhibit long germ-band development, and co-association with the preserved remains of post-embryonic segmented larvae that exhibit bilaterally arranged pairs of anatomical domains [4]. There has been some debate about the pattern of segmentation [5] exhibited by *Pseudooides*, but this interpretation, as among the oldest body fossil evidence of ecdysozoans, has been generally accepted. An early attempt to characterize the morphology of the germ band using synchrotron radiation X-ray tomographic microscopy (SRXTM) observed that the marginal, median and inter-metameric furrows were incompletely preserved, extending only to a shallow depth within the embryos, not enclosing the metameres ventrally [5]. In an attempt to better characterize the anatomy, development, affinity and therefore the evolutionary significance of *P. prima*, we recovered additional material which we subjected to SEM and SRXTM characterization. Our results reveal that the ‘germ band’ of *Pseudooides* develops into an aperture that unfolds along the midline furrow and ‘metamere’ boundaries, resulting in a 10-sided, bilaterally symmetrical theca with a concave base. This stage mirrors the prototheca of the co-occurring *Hexaconularia*, a hexangulaconulariid allied to the conulariids [6], conventionally interpreted as coronate scyphozoan cnidarians [7]. Our phylogenetic analyses, which build on our interpretation of *Pseudooides*, support a scyphozoan affinity, allying it with a clade of direct-developing scyphozoans exhibiting diverse symmetry. This suggests that the largely tetraradial and indirect-developing extant Cnidaria are a vestige of a broader early Palaeozoic diversity.

## 2. Material and methods

Studied material was recovered from the early Cambrian (Fortunian, Terreneuvian) Kuanchuanpu Formation at the Shizhonggou Section in Ningqiang County, Shaanxi Province, China. At Shizhonggou, the stratigraphic distribution of *P. prima* falls fully within the *Anabarites trisulcatus–Protohetzina anabarica* Assemblage Biozone [8], falling within the span of 537–532 Ma [9]. The samples were processed by routine etching with approximately 10% technical grade acetic acid following Müller [10] and were then separated from the insoluble residue by manual picking under a binocular microscope. Collected specimens were analysed using environmental scanning electron microscopy (ESEM) and SRXTM [5]. We examined 19 specimens of *Pseudooides* using SRXTM at the X02DA TOMCAT beam line of Swiss Light Source, Paul Scherrer Institute, Villigen, Switzerland. Twelve of the specimens preserve internal structure and these were analysed using the AVIZO 8.0 software. The illustrated figures were assembled using Adobe Photoshop CS. All figured specimens are deposited in the Geological Museum of Peking University (GMPKU), Beijing.

The phylogenetic dataset was compiled and designed to test the phylogenetic relationships of *Pseudooides* and other seemingly related taxa. Previous studies have concluded that some of these early Cambrian fossils may represent crown cnidarians [11]. Taxon sampling was guided by the aim of evaluating their affinities within the diversity of this phylum. The dataset sampled six out-groups: three representative bilaterians and the three non-bilaterian phyla (Porifera, Placozoa and Ctenophora). Characters were derived from previous studies addressing apomorphies that are general to metazoans [12,13], ctenophores [14], and medusozoan [15,16] and anthozoan [17,18] cnidarians; characters describing medusozoans’ tube morphologies are derived from Mendoza-Becerril *et al.* [19]. We followed the original coding for all non-modified characters. Simplified characters (three states converted into binary absence/presence) were coded as ‘present’ for the different morphologies when they were not absent. The resulting matrix contains 47 taxa coded for 214 characters.

The phylogenetic analysis was undertaken using MrBayes (Windows v. 3.2) under the Mkv model (invgamma rates, variable coding). The analysis was run twice to assess convergence. Each replicate had four chains running for 10 million generations. Both runs reached convergence (based on criteria established in the MrBayes manual) before the 10 million generations and produced the same topology albeit with minor differences in posterior probabilities.

## 3. Results

**Figure 1.**
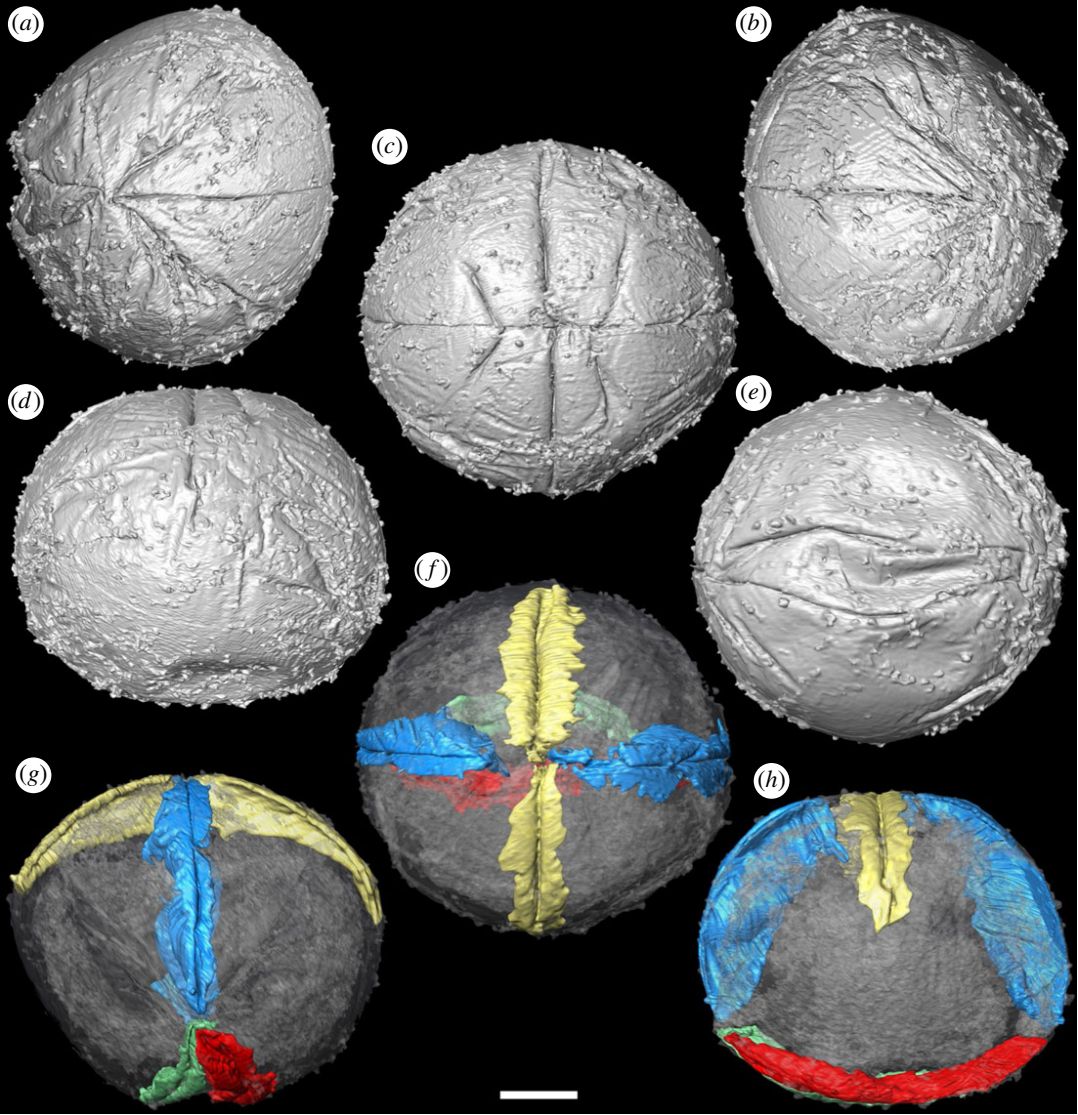
Tomographic surface models of *Pseudooides* specimens preserving the initial stages of delineation of the ‘germ band’ (GMPKU3100). (*a–e*) SRXTM surface renderings. (*a*) Left, (*b*) central and (*c*) right views of the ‘germ band’; (*d*) lateral view, with ‘germ band’ top and centre; (*e*) opposing pole of the specimen. (*f–h*) SRXTM rendering of the longitudinal furrow (blue), transverse furrow (gold) and the basal folds (green, red): (*f*) central view of the ‘germ band’; (*g*) lateral view of the longitudinal furrow, (*h*) lateral view of the transverse furrow. Scale bar, (*a–h*) 52.5 µm. (Online version in colour.)

**Figure 2.**
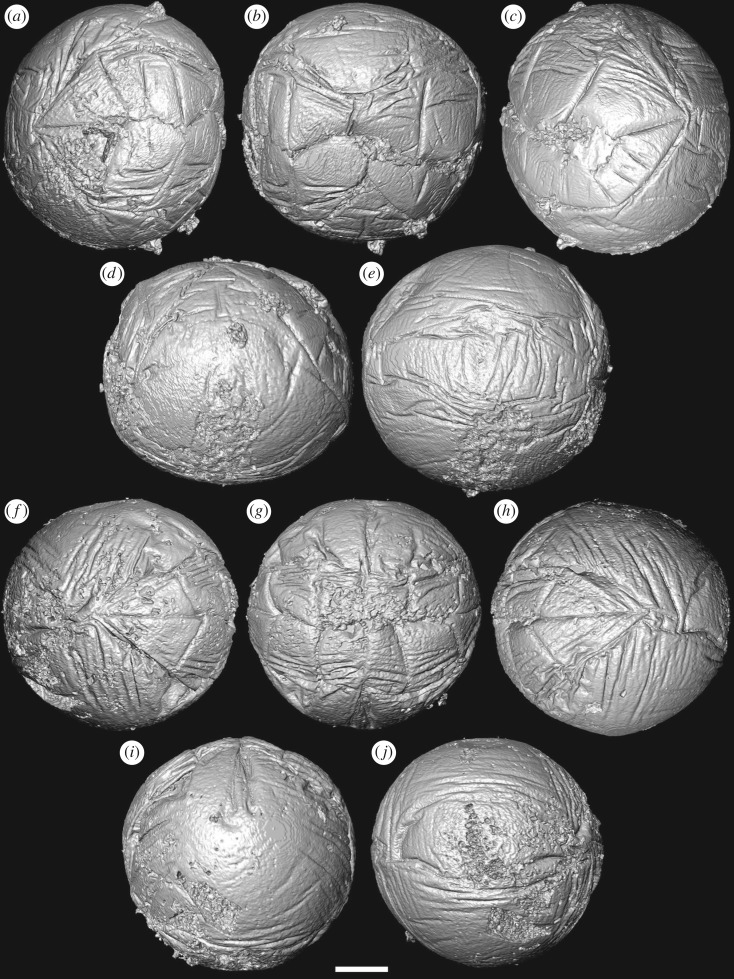
Tomographic surface models of *Pseudooides* specimens preserving the ‘germ band’. (*a–e*) SRXTM renderings of specimen preserved with complete ‘germ band’ with a pinch at mid-length (GMPKU3091). (*a*) Left, (*b*) central and (*c*) right views of ‘germ band’; (*d*) lateral view of the specimen, with ‘germ band’ top and centre; (*e*) opposing pole of the specimen. (*f–j*) SRXTM renderings of specimen with preserved complete ‘germ band’ without a pinch at mid-length (GMPKU3099). (*f*) Left, (*g*) central and (*h*) right views of the ‘germ band’; (*i*) side view of the specimen, with the ‘germ band’ top and centre; (*j*) opposing pole of the specimen. Scale bar, (*a–e*) 81 µm, (*f–j*) 66 µm.

**Figure 3.**
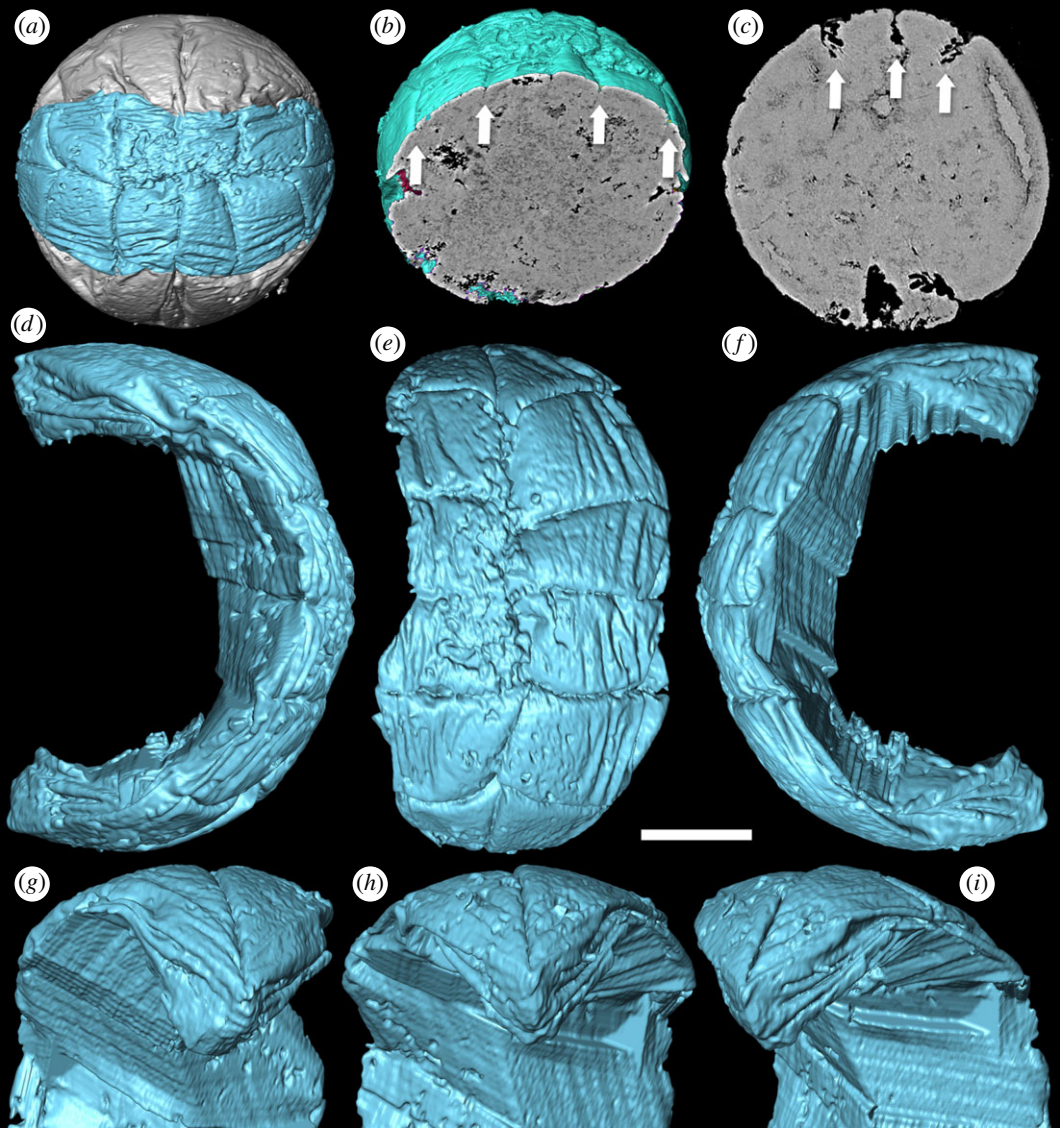
*Pseudooides* specimens with furrows of the ‘germ band’. (*a,b,d–i*) SRXTM renderings of the specimen with delicate ‘germ band’ furrows preserved (GMPKU3099). (*a*) SRXTM renderings of the complete ‘germ band’ (indigo-blue); (*b*) the virtual section shows that the ‘inter-metameric’ furrows are largely surface features (arrows); (*d*) left, (*e*) central and (*f*) right views of the marginal furrows of the ‘germ band’ with surrounding material removed; (*g*) left, (*h*) central and (*i*) right close-ups of the tip end of the ‘germ band’. (*c*) The virtual section positioned between the pinch and one end of the ‘germ band’ shows the depth of the marginal furrow and the median furrow (arrows, GMPKU3094). Scale bar, (*a,b*) 100 µm, (*c*) 93 µm, (*d–f*) 62 µm, (*g–i*) 52 µm. (Online version in colour.)

**Figure 4.**
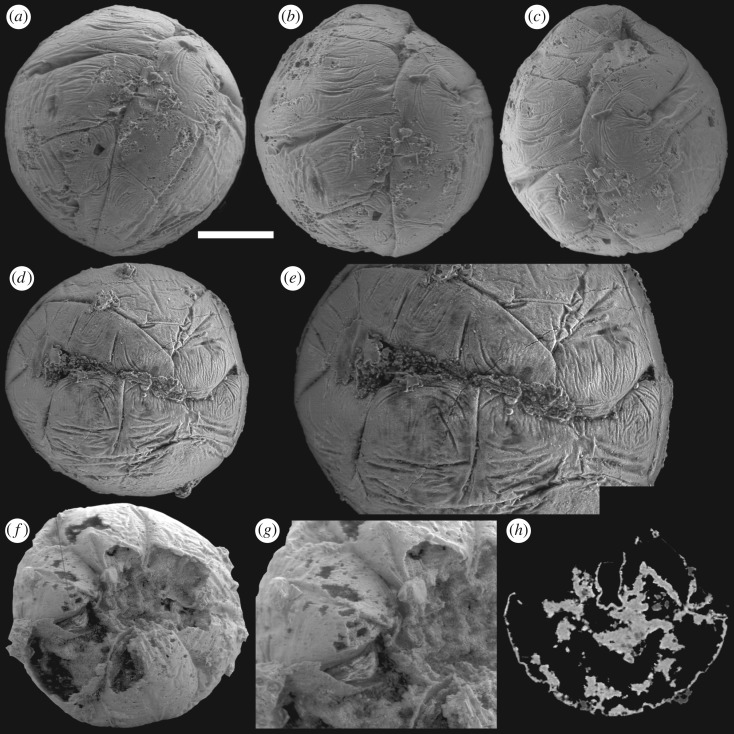
*Pseudooides* specimens display the unfolding of the furrows. (*a–c*) Specimen with the unfolding median furrow (GMPKU3096). (*a*) The folded end of the ‘germ band’; (*b*) the middle part of the ‘germ band’ showing the median furrow starting to unfold; (*c*) the unfolding end of the ‘germ band’ with paired ‘metameres’ separated. (*d,e*) Specimen with a separated median furrow (GMPKU3091). (*d*) Front view of the ‘germ band’; (*e*) close-up of (*d*). (*f–h*) Specimen with a broken ‘germ band’ surface (GMPKU3104). (*f*) SEM image of the specimen, grey line marking the position of the virtual section of (*h*); (*g*) close-up of the exposed median furrow; (*h*) virtual section showing the extension of the putative ectoderm layer within the embryo. Scale bar, (*a,b*) 100 µm, (*c*) 105 µm, (*d*) 123 µm, (*e*) 78 µm, (*f*) 115 µm, (*g*) 70 µm, (*h*) 124 µm.

Our SRXTM analyses reveal that the furrows between the adjacent metameres are not incompletely preserved but, rather, extend continuously as a single folded layer of presumed ectoderm (figures 1–4). The ectoderm within the marginal furrows exhibits repeated longitudinal folding (figures 1*f–h* and 3*b–i*); at its deepest, at the ends of the ‘germ band’ (figure 3*c–i*), the furrow ectoderm exhibits up to five folds that converge, reducing in number and becoming more tightly folded, as the furrow approaches the ‘pinch’ at mid-length (figure 3*g–i*). The ‘inter-metameric’ furrows are largely surface features that have minimal depth (figure 3*b*), extending laterally before the delineation of the ‘germ band’ (cf. figures 1*a–d* and 2*a–d*). In most specimens, the median longitudinal furrow is narrow in width (approx. 2 µm) and varies in depth between specimens due to preservational differences (figures 1*f–h* and 3*c*). Maximally, the median furrow extends 71 µm, approximately vertically into the embryo, converging at depth from an average width of 2 µm at the surface. The digital cast of the furrow is smooth and has open convolutions that diminish with depth (figures 1*f–h* and 3*a*). Internally, the embryo is otherwise composed of homogeneous calcium phosphate mineral (figure 3*b,c*), reflecting diagenetic cements filling voids remaining after the initial mineralization of biological structures and decay of organics; this void-filling phase is not apparent in all specimens (e.g. figure 4*h*).

The absence of a distinct central surface to the ‘metameres’ is not incompatible with their interpretation as comprising a germ band. However, the deep extension of the median furrow depth is not expected because it suggests that this longitudinal border is not equivalent to the transverse ‘metamere’ boundaries. This distinction is borne out in specimens in which the median furrow shows greater degrees of expansion, and paired ‘metameres’ are separated (figure 4), in some specimens revealing a finite margin to the putative ectoderm layer (figure 4*a–e*). The unfolding of marginal furrows in some specimens, especially at both ends of the ‘germ band’, is also observed (figure 4*f–h*). These suggest that the putative ‘germ band’ is, rather, an aperture onto the interior of the embryo, and the transverse and marginal ‘metamere’ furrows represent foldings of this aperture. In a partially broken specimen (figure 4*f–h*), the ventral margin of the median furrow has evaginated and its distinct margins are exposed; the folded triangular poles of this aperture are no longer adpressed to, and aligned with, the spherical surface of the embryo.

The abapertural pole of the embryos often exhibits a longitudinal concavity in the ectoderm, up to 20% radius depth, with marginal wrinkles or furrows, and a scratch with diameter ranging from 30 to 100 µm in the middle, all aligned parallel to the aperture (figures 1*e–h* and 2*e,j*). These structures are not the same as a previously described blastopore [5], which is a preservational artefact and not an original biological structure. This abapertural concavity is associated with the lateral flattening of the embryo, and although it could be a taphonomic feature, its alignment with the long axis of the aperture suggests that it is biological and reflects development.

**Figure 5.**
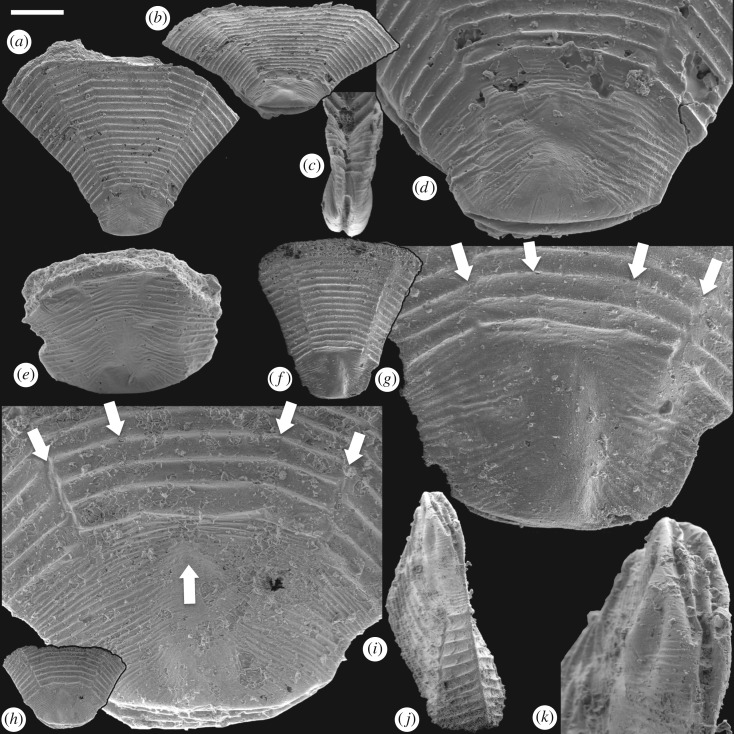
SEM images of post-embryonic ‘*Hexaconularis sichuanensis*' stages of *P. prima*. (*a–d*) GMPKU3132. (*a*) Lateral view of the polyp theca; (*b*) oblique view of the theca showing the concave base of the retained embryonic stage; (*c*) side view of the concave base of the retained embryonic stage; (*d*) close-up of the retained embryonic stage showing the four lateral cuticular folds represented as furrows in the ‘germ band’ of the embryo. (*e*) GMPKU3133; lateral view of the retained embryonic stage. (*f–g*) GMPKU3134. (*f*) Lateral view of the polyp theca; (*g*) close-up of the retained embryonic stage with the four lateral cuticular folds (arrows) reflecting the furrows in the ‘germ band’ of the embryo. (*h–k*) GMPKU3135. (*h*) Lateral view of the polyp theca; (*i*) close-up of the retained embryonic stage with the four lateral cuticular folds (down arrows) reflecting the furrows and the pinch (up arrow), respectively, in the ‘germ band’ of the embryo. Scale bar, (*a,b*) 409 µm, (*c*) 169 µm, (*d*) 119 µm, (*e*) 132 µm, (*f*) 406 µm, (*g*) 104 µm, (*h*) 444 µm, (*i*) 95 µm, (*j*) 189 µm, (*k*) 73 µm.

**Figure 6.**
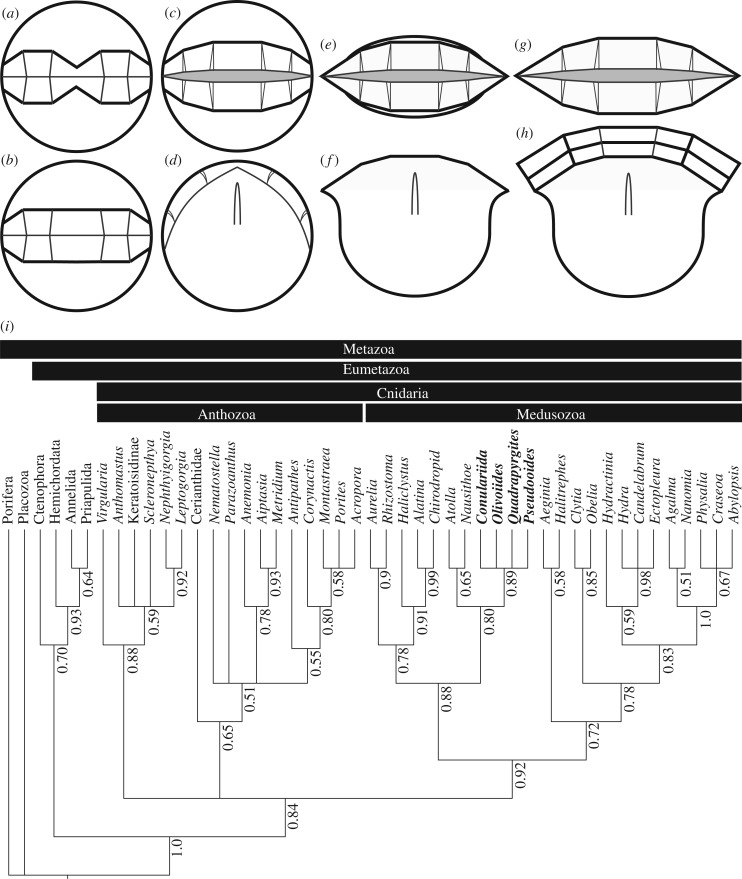
Development and phylogenetic affinity of *P. prima*. (*a–h*) Diagrammatic characterization of embryonic (*a–d*) and post-embryonic (*e–h*) development. (*a*) Delineation of the aperture; (*b*) opening of the central pinch of the aperture; (*c–d*) apertural and lateral view, respectively, at the first opening of the aperture; (*e–f*) apertural and lateral view, respectively, at the unfolding of the lateral apertural extremes and lateral compression of the original spherical embryonic tissue; (*g–h*) apertural and lateral view, respectively, of the first stages of post-embryonic thecal growth at the apertural margin. (*i*) Phylogenetic relationships among living Cnidaria and the direct-developing medusozoans considered here, including *P. prima*, based on a Bayesian phylogenetic analysis of phenotypic characters. Codings for *Pseudooides* are based on the interpretation presented in this study. *Pseudooides*, conulariids and the olivooids are resolved as a clade closely related to the living coronate scyphozoans, including *Nausithoe*. Numbers adjacent to nodes represent their posterior probabilities.

This pattern of embryonic development of *Pseudooides* is compatible with the post-embryonic development of the putative scyphozoan cnidarian *Hexaconularia* (figure 5), which occurs in the same samples as *Pseudooides* [8]. The earliest developmental stages in our collections of *Hexaconularia*, preserved as the base of the polyp theca, are comparable with a flattened *Pseudooides* embryo (compare figures 2 and 5; see also figure 6*a–h*). The prototheca of *Hexaconularia* has an aperture-parallel aboral furrow or concavity (figure 5*b–d,i–k*) and marginal wrinkles (figure 5*d,e,j,i*). These wrinkles occur in far greater number in the prototheca of *Hexaconularia* than in the embryo of *Pseudooides*; we infer this difference to be associated with the flattening of the spherical embryo of *Pseudooides* to the bilaterally compressed theca of *Hexaconularia*. The aperture has acute ends (figure 5*c,j,k*), reflecting the unfolding of the extreme ends of the invaginated embryonic aperture, and thus it represents the widest point of what is conventionally interpreted as the polyp theca. The wrinkled appearance of the *Pseudooides* embryos and the concave abapical region of the *Hexaconularia* thecae are identical, reflecting a thin cuticle. The two dominant, and two more subtle, bilateral pairs of ridges aligned with the ab-adapertural axis of the *Hexaconularia* theca (figure 5*g,i*) reflect the four transverse ‘metameric’ furrows of the *Pseudooides* ‘germ band’. Together with the ridges that define the ‘corners’ at the lateral extremes of the *Hexaconularia* aperture (figure 5*c,j*) and *Pseudooides* ‘germ band’, these paired ridges comprise ten [20], rather than the conventional interpretation of six [6], faces comprising the *Hexaconularia* theca. The aperture of the embryo actually begins with five transverse furrows, circumscribing six paired domains, including a central furrow that extends approximately half of the circumference of the embryo (figure 1). However, this long central transverse furrow is subsequently lost as the central domain of the aperture unfolds, leading to the ‘pinched’ stage (figures 2*a–e* and 6*a*). No vestige of this central transverse furrow remains in the adult theca.

The lateral axial depression in the abapical division of the *Hexaconularia* theca [21] (figure 5*d,g,h*) reflects the folding of the ectoderm associated with the transverse ‘pinch’ in the invaginated aperture of the *Pseudooides* embryo [4,5] (figure 2*a–d,i*). Later development is dominated by the extension and expansion of the theca, which appears to have occurred episodically, through the extension of the aperture, punctuated by tranverse ridges. The division of the theca into distinct abapical and apical regions has been highlighted previously, suggested to reflect embryonic and post-embryonic stages of development [21]. Our demonstration of a developmental link to *Pseudooides* confirms this suggestion.

In an attempt to resolve the phylogenetic affinity, and therefore the evolutionary significance of these direct-developing metazoans, we compiled a cladistic dataset of phenotypic characters, building on previous attempts [15,16] but expanded to encompass extant anthozoan and medusozoan diversity, as well as ctenophore, sponge, placozoan and bilaterian out-groups. We subjected this to Bayesian phylogenetic inference using the Mk + G model (controlling for ascertainment bias [22]) in MrBayes [23]. The majority consensus of the posterior distribution of trees is surprisingly well-resolved, given that it is based on a phenotype dataset, with good support for many clades and conforming largely with existing hypotheses of relationships for the extant taxa (figure 6*i*). Within this scheme, *Pseudooides* is resolved within a clade with the extinct olivooids and Conulariida, sister to the extant scyphozoans *Nausithoe* and *Atolla*, nested among a broader clade of scyphozoans and cubozoans (figure 6*i*).

## 4. Discussion

The discovery that *Pseudooides* and *Hexaconularia* represent distinct developmental stages of the same organism requires some remedial taxonomy; *Pseudooides* has priority and we recognize *Hexaconularia* as a junior synonym. This mirrors the synonymy of *Olivooides* and its junior synonym *Punctatus* [24], which are embryonic and post-embryonic stages of development of the same organism [25]. The previous interpretation of *Pseudooides* as an ecdyszoan was based largely on the interpretation of a germ band [4,5], which we reinterpret as a developing aperture. It was also originally based on co-association with fragments of an arthropod-like segmented and appendage-bearing organism or organisms [4]; we consider this association coincidental [26].

A cnidarian affinity for *Hexaconularia* has been justified principally through affinity to the extinct conulariids [6,21,27] that have long been compared with coronate scyphozoan cnidarians [7,28]. However, a more convincing case can be made based on similarity of development in *Pseudooides* and *Olivooides*, which also develops directly from embryo to polyp. In its case*, Olivooides* retains the stellate periderm of the embryo at the aboral apex of the post-embryonic theca [11,20,24,25], from which the theca expands through episodic release of periderm annulae at an aperture that is interpreted to have developed seasonally [11], akin to the operculum of the extant coronate scyphozoan *Nausithoe* [29]. There is evidence that this mode of direct development is manifest also in other hexangulaconulariids, in particular *Arthrochites* whose theca exhibits a morphologically distinct apex [6], presumably betraying retention of periderm from an embryonic stage of development [21]. A similar structure, the schott [30], occurs in at least some extinct conulariids. Van Iten *et al.* [21] have argued that its genesis is distinct, as a repair response to broken holdfasts.

Regardless, the conulariid schott suggests an equivalent mode of life to hexanguloconulariids and olivooids, as a sediment- or mat-sticker, rather than fused to the substrate [24]. This ecological strategy may be a consequence of the direct mode of development adopted by these scyphozoans. However, it may also reflect the stable nature of the mat-bound substrates that obtained in shallow marine environments in the early Cambrian period [31]. Thus, though direct development from embryo to polyp is uncommon among extant [32] cnidarians, this may be a consequence of the Cambrian substrate revolution. Further, the peculiar preservation of embryonic and post-embryonic developmental stages may also reflect their predisposition to fossilization through the precocial development of cuticular periderm within the embryo.

The results of our phylogenetic analysis suggest that *Pseudooides* is a member of a broader clade of direct-developing scyphozoan cnidarians, with a life history that is quite distinct from their living relatives. It is also a clade that exhibits an extraordinary level of body-plan symmetry, encompassing tetraradial, pentaradial and pseudo-hexa-radial forms. Together, these factors suggest that the life history modes and organizational symmetry of extant medusozoans are a relict of a broader diversity apparent early in their evolutionary history. The early Cambrian Fortunian age of these fossils combined with their total-group Coronata affinity provides intrinsic evidence for an extensive pre-Phanerozoic history to the cnidarian and eumetazoan crown and total groups, compatible with claims of Ediacaran fossil cnidarians [33] and molecular clock estimates for the origin of these clades [34].

## 5. Systematic palaeontology

Phylum CNIDARIA Hatschek, 1888

Class SCYPHOZOA Götte, 1887

Order ?CONULARIIDA Miller and Gurley, 1896

Family HEXANGULACONULARIIDAE He, 1987

Genus *Pseudooides* Qian, 1977

*Pseudooides* Qian [2], p. 217

*Hexaconularia* (nomen nudum) Yang, He and Deng [35], p. 95

*Hexaconularia* He and Yang [36], p. 35

Type species. *Pseudooides prima* Qian [2]

*Emended diagnosis*. Organism composed of two distinct sections; the lenticular apex of embryonic tissue and the tapered, highly compressed abapical region of post-embryonic tissue. The apical region is relatively smooth with closely spaced ridges, while the abapical tissue is coarse with regular transverse ridges. The embryo is an approximately 0.3 mm sphere in which a longitudinal aperture develops, the margins of which are folded, giving the appearance of six paired and bilaterally arranged domains that prefigure the arrangement of the architecture of the adult, and taper to a point at both ends.

*Description*. The genus forms highly compressed (transv.) and tapered skeleton or theca with a lenticular apex of embryonic tissue and much larger abapical post-embryonic tissue with the transverse cross section elongate with ten sides arranged tetraradially. The embryonic tissue is with two gently convex faces and a gently curved broad tip with a terminal furrow; faces of the embryonic tissue crossed by fine, closely spaced, relatively irregular transverse ridges. The post-embryonic tissue is with an open aperture and 10 faces exhibiting characteristic transverse ridges.

*Remarks*. A post-embryonic stage of the organism, in which the aperture of the embryo is fully open, but post-embryonic thecal tissues have not yet developed, has yet to be recovered. However, such absence is remedied by the homologous structures observed at embryonic and post-embryonic developmental stages, such as the terminal furrow and surface ridges on the apical tissue, as well as the ten faces (metameres of the embryonic stage) and the transverse ridges on the abapical tissue. The embryonic stage of the organism is distinctive from other fossil embryos for its relatively smaller size (approx. 300 µm) and typical ‘8’-shaped wrinkles on the abapical tissue.

*Pseudooides prima* Qian [2]

Figures 1–5

*Pseudooides prima* Qian [2], p. 217 pl. 2, fig. 26.

*Hexaconularia multicostata* (nomen nudum) Yang, He and Deng [35], p. 95, pl. 3, figs 6, 12; He [37], p. 10, 12.

*Hexaconularia nanjiangensis* (nomen nudum) Yang, He and Deng [35], pp. 94–95, pl. 3, fig. 8; He [37], p. 12.

*Hexaconularia sichuanensis* (nomen nudum) Yang, He and Deng [35], p. 94, pl. 3, fig. 10a,b; He [37], p. 12.

*Hexaconularia xinliensis* (nomen nudum) Yang, He and Deng [35], p. 95, pl. 3, fig. 11a,b; He [37], p. 9, 10, 12.

*Hexaconularia* sp. (nomen nudum) Yang, He and Deng [35], pl. 3, fig. 7.

*Hexaconularia sichuanensis* He and Yang [36], pp. 35–36, pl. 4, figs 5, 6.

*Hexaconularia nanjiangensis* He and Yang [36], p. 36, pl. 4, fig. 2.

*Hexaconularia breviculus* He and Yang [36], p. 36, pl. 4, figs 7–9.

*Hexaconularia xinliensis compressiformis* He and Yang [36], p. 37, pl. 5, fig. 1.

*Hexaconularia xinliensis crassiformis* He and Yang [36], p. 37, pl. 5, figs 3, 4.

*Hexaconularia bellulus* He and Yang [36], p. 37, pl. 5, figs 8–10.

*Hexaconularia bellulus compressiformis* He and Yang [36], p. 38, pl. 5, fig. 2.

*Hexaconularia longelus* He and Yang [36], p. 38, pl. 5, figs 11, 12.

*Hexaconularia multicostata* He and Yang [36], pl. 5, figs 5–7.

*Barbitositheca ansata* Brasier and Singh [38], p. 335, 337, figs 7.1–7.3.

?*Conulariella quadrata* (nomen nudum) He [37], p. 9, 11, 13.

*Hexangulaconularia* cf. *formosa* Brasier and Singh [38], p. 337, figs 7.4–7.6.

*Hexangulaconularia formosa* Qian, 1989, pp. 161–162, pl. 28, figs 7–9; text-fig. 32.

*Hexangulaconularia xinliensis* (Yang *et al*.); Qian [3], pp. 162–163, pl. 10, figs 12, 13; pl. 28, fig. 6.

*Pseudooides prima* Qian [2]; Qian [3], p. 262, pl. 71, fig. 6.

*Hexaconularia sichuanensis* He and Yang [36]; Conway Morris and Chen [6], pp. 400–401, figs 12.8–12.14. (cum. syn.).

Embryo type B; Steiner *et al*. [8], p. 266, fig. 4.4.

*Hexaconularia sichuanensis* He and Yang [36]; Steiner *et al*. [8], p. 270, figs 8.1–8.2.

*Pseudooides prima* Qian [2]; Steiner *et al*. [4], p. 834, fig. 2I–O (partim, cf. figs A-H; non figs 2P-S).

*Pseudooides* Qian [2]; Donoghue and Dong [26], p. 89, fig. 3c–e.

*Pseudooides prima* Qian [2]; Donoghue *et al*. [5], p. 682, fig. 3.

*Hexaconularia sichuanensis* He and Yang [36]; Van Iten *et al*. [21], pp. 192–196, figs 1, 2.

*Hexaconularia sichuanensis* He and Yang [36]; Steiner *et al*. [20], p. 104, figs 7.13–7.16, 7.19–7.21.

*Pseudooides prima* Qian [2]; Donoghue *et al*. [1], p. 54, fig. 3.5a–c.

*Hexaconularia sichuanensis* He and Yang [36]; Zhang *et al*. [39], p. 3, fig. 2.9.s.

*Diagnosis*. As for the genus.

*Description*. In the pre-hatching embryonic stage, the apical tissue occupies the major part of the embryo; the surface of the apical tissue is mostly smooth, with fine, closely spaced ridges along the boundary with the abapical tissue; the aboral pole of the embryo is characterized by a transverse terminal furrow. The abapical tissue has a highly wrinkled surface appearance usually with a central pinch—a vestige of the expansion of the centre of the aperture in late embryonic development; 10 apertural domains, separated by the intermediate furrows (not including the pinched centre), are homologous to 10 faces of the post-embryonic tissue; the median furrow represents the aperture of the organism.

After hatching, the apical tissue is flattened, forming a lenticular shape and, with the expansion of the theca at the aperture, occupying an increasingly subordinate part of the organism; the surface ornamentation deforms with the compression of the embryo, but remains mostly unchanged. The abapical tissue is unfolded and stretches after hatching. Specifically, in the embryonic stage, furrows at tapering ends of the aperture evaginate, forming the tapered transitional region of the post-embryonic stage between the abapical and apical tissues; the abapical tissue gives rise to the tapered abapical post-embryonic tissue with ten faces and an aperture, exhibiting regular trochoidal transverse ridges.

*Remarks*. The image of the holotype shows the typical 8-shaped structure of the embryonic developing aperture of the species, yet provides little insight into post-embryonic development. Most of the existing taxonomy has been based on the adult specimens, with regard to the morphological differences of the apical or abapical regions, such as the angle and the number of ridges of the apicial region, the angle of the abapical region, and the shape and aspect ratio of the transverse section of the abapical region. In the light of the developmental insights afforded by our data, we consider these differences to be more likely to represent individual or preservation differences, rather than distinguishing characteristics of distinct taxa.

## 6. Conclusion

The early Cambrian *Pseudooides prima* has been described as among the earliest records of ecdysozoans, based on embryonic and post-embryonic stages of development. Based on new collections, we characterize the embryonic stages tomographically, using synchrotron radiation X-ray computed tomography. We show that the ‘germ band’ of *P. prima* embryos separates along its mid axis during development, with the transverse furrows between the ‘somites’ unfolding into the polar aperture of the ten-sided theca of *Hexaconularia sichuanensis*, conventionally interpreted as a scyphozoan cnidarian. Co-occurring post-embryonic remains of ecdysozoans are unrelated. Direct development in *P. prima* parallels the co-occuring olivooids *Arthrochites*, *Olivooides* and *Quadrapyrgites,* and Bayesian phylogenetic analysis of a novel phenotype dataset indicates that, despite differences in their symmetry, the hexangulaconulariids and olivooids comprise a clade of scyphozoan medusozoans, with *Arthrochites* and conulariids, most of which exhibit direct development from embryo to thecate polyp. Like some conulariids, these coronates lived embedded within, rather than fused to, sedimentary substrates, presumably a consequence of their unique mode of direct development. The affinity of hexangulaconulariids to extant scyphozoan medusozoans indicates that the prevalence of tetraradial symmetry and indirect development is a vestige of a broader spectrum of body-plan symmetries and developmental modes that was manifest in their early Phanerozoic counterparts.

## Supplementary Material

Character_descriptions.docx

## Supplementary Material

Pseudooidesmatrixfinal.txt

## Data accessibility

The supporting tomographic and phylogenetic datasets are available from the University of Bristol data repository (data.bris) at https://doi.org/10.5523/bris.3vd8kczvuzo3y2nrn6n8m6l2y4 and Figshare, respectively.
