# Supplementary material for: The early Cambrian fossil embryo Pseudooides is a direct-developing cnidarian, not an early ecdysozoan
Source: Proc Biol Sci. 2017 Dec 13;284(1869):20172188. doi: 10.1098/rspb.2017.2188 (PMC5745419; doi:10.1098/rspb.2017.2188)
Supplement: Character_descriptions.docx [file rspb20172188supp1.docx]

**Character Descriptions**

1. Outer epithelia with septate or tight junctions: (0, absent; 1, present). Character 1 in Nielsen et al. 1996.
2. Nerve cells with chemical synapses: (0, absent; 1, present). Character 2 in Nielsen et al. 1996.
3. Basement membrane: (0, absent; 1, present). Character 3 in Nielsen et al. 1996.
4. Synapses with acetylcholine: (0, absent; 1, present). Character 4 in Nielsen et al. 1996.
5. Gap junctions: (0, absent; 1, present). Character 5 in Nielsen et al. 1996.
6. Striated ciliary rootlets: (0, absent; 1, present). Character 6 in Nielsen et al. 1996.
7. Monociliate epithelia: (0, absent; 1, present). Character 7 in Nielsen et al. 1996.
8. Multiciliate epithelia: (0, absent; 1, present). Character 8 in Nielsen et al. 1996.
9. General body cuticle with collagen: (0, absent; 1, present). Character 9 in Nielsen et al. 1996.
10. Chitin present in epithelial organs: (0, absent; 1, present). Character 10 in Nielsen et al. 1996.
11. General body cuticle with chitin: (0, absent; 1, present). Character 11 in Nielsen et al. 1996.
12. Oral region with chitin spines: (0, absent; 1, present). Character 12 in Nielsen et al. 1996.
13. Cuticle moulted: (0, absent; 1, present). Character 13 in Nielsen et al. 1996.
14. Trunk without cuticle, epithelia with intra-cellular skeletal lamina: (0, absent; 1, present). Character 14 in Nielsen et al. 1996.
15. Collar complexes: (0, absent; 1, present). Character 15 in Nielsen et al. 1996.

**REPRODUCTION AND DEVELOPMENT**

1. Gametic meiosis: (0, absent; 1, present).Ax1995.
2. Organized gonads: (0, absent; 1, present). Character 16 in Nielsen et al. 1996.
3. Gonads with separate gonoducts: (0, absent; 1, present). Character 17 in Nielsen et al. 1996.
4. Gametes pass through coelom and metanephridia: (0, absent; 1, present). Character 18 in Nielsen et al. 1996.
5. Sperm with single compact acrosome: (0, absent; 1, present). Character 19 in Nielsen et al. 1996.
6. Sperm with mitochondria between the nucleus and the flagellum: (0, absent; 1, present). Based on several sources. This condition is seen in cnidarians and bilaterians but not in ctenophores or sponges. Character state not known in Placozoa. Potential synapomorphy of Planulozoa.
7. Spiral cleavage with 4-d mesoderm: (0, absent; 1, present). Character 20 in Nielsen et al. 1996.
8. Larva with ciliated apical sense organ: (0, absent; 1, present). Character 21 in Nielsen et al. 1996.
9. Larvae or adult with downstream-collecting ciliary bands of compound cilia on multiciliate cells: (0, absent; 1, present). Character 22 in Nielsen et al. 1996.
10. Larvae or adult with upstream-collecting ciliary bands with single cilia on monociliate cells: (0, absent; 1, present). Character 23 in Nielsen et al. 1996.
11. Larval apical organ lost at metamorphosis and a new brain develops from a ventral invagination in Endoderm and from an invagination of uncertain position in Ectoderm: (0, absent; 1, present). Character 24 in Nielsen et al. 1996.
12. Sexual condition: (0, hermaphroditic; 1, gonochoric). Character 19 in Marques & Collins 2004.

**ARCHITECTURE**

1. Body with ectoderm and endoderm: (0, absent; 1, present). Character 25 in Nielsen et al. 1996.
2. Body with ectoderm, endoderm, and mesoderm: (0, absent; 1, present). Character 26 in Nielsen et al. 1996.
3. Mesoderm formed from 4d-cell, from the blastopore ridge, or as ectomesoderm: (0, absent; 1, present). Character 27 in Nielsen et al. 1996.
4. Mesoderm formed from archenteron: (0, absent; 1, present). Character 28 in Nielsen et al. 1996.
5. Body segmented with serially repeated organs developed from 4d-mesoderm (or ectomesoderm): (0, absent; 1, present). Character 29 in Nielsen et al. 1996.
6. Body with successively added segments developed from a teloblastic growth zone: (0, absent; 1, present). Character 30 in Nielsen et al. 1996.
7. Body with segmented longitudinal musculature developed from rows of mesodermal pockets from the archenteron: (0, absent; 1, present). Character 31 in Nielsen et al. 1996.
8. Body archimeric: (0, absent; 1, present). Character 32 in Nielsen et al. 1996.
9. Blastopore remains as mouth/anus: (0, absent; 1, present). Character 33 in Nielsen et al. 1996.
10. Mouth terminal, pharynx radial: (0, absent; 1, present). Character 34 in Nielsen et al. 1996.
11. Mouth on tip of inversible introvert: (0, absent; 1, present). Character 35 in Nielsen et al. 1996.
12. Introvert with spines, teeth and scalids: (0, absent; 1, present). Character 36 in Nielsen et al. 1996.
13. Non-inversible mouth cone with cuticular ridges and spines: (0, absent; 1, present). Character 37 in Nielsen et al. 1996.
14. U-shaped pharyngeal gill slits with collagenous skeleton: (0, absent; 1, present). Character 38 in Nielsen et al. 1996.
15. Chorda: (0, absent; 1, present). Character 39 in Nielsen et al. 1996.
16. Notochord: (0, absent; 1, present). Character 40 in Nielsen et al. 1996.
17. Endostyle: (0, absent; 1, present). Character 41 in Nielsen et al. 1996.
18. Food manipulated with modified limbs: (0, absent; 1, present). Character 42 in Nielsen et al. 1996.
19. Limbs articulated with intrinsic muscles: (0, absent; 1, present). Character 43 in Nielsen et al. 1996.
20. Oral-aboral axis: (0, absent; 1, present). Defined as the primary axis of body symmetry in animals. Plesiomorphically present in all animals, uncertain inplacozoans.
21. Dorsoventral axis:(0, absent; 1, present). The secondary axis of symmetry that defines bilateral symmetry. Present in bilaterians and apparently also in cnidarians.
22. Mesoglea: (0, absent; 1, present). This character includes the coelenterate mesoglea as well as the poriferanmesohyl.
23. Mesoglea: (0, non-cellular; 1, cellular). Character 4 in Marques & Collins 2004.
24. Ectodermal pharynx: (0, absent; 1, present). Present in anthozoans, protostomes and ctenophores.

**NERVOUS SYSTEM**

1. Adult brain derived from or associated with larval apical organ/apical pole: (0, absent; 1, present). Character 44 in Nielsen et al. 1996.
2. Ventral longitudinal nerves paired or secondarily fused (seen during ontogeny):(0, absent; 1, present). Character 45 in Nielsen et al. 1996.
3. Dorsal nerve concentration/brain behind apical organ/apical pole: (0, absent; 1, present). Character 46 in Nielsen et al. 1996.
4. Dorsal longitudinal nerve cord: (0, absent; 1, present). Character 47 in Nielsen et al. 1996.
5. Brain collar-shaped, consisting of anterior and posterior rings with perikarya separated by a ring of neuropile: (0, absent; 1, present). Character 48 in Nielsen et al. 1996.
6. Proto-, deuto- and tritocerebrum: (0, absent; 1, present). Character 49 in Nielsen et al. 1996.
7. Nervous system composed of a diffuse nerve net: (0, absent; 1, present). This character represents the difference between a nervous system composed of nerves and ganglions and the nets seen in coelenterates.

**CIRCULATORY AND EXCRETORY SYSTEMS**

1. Haemal system, i.e. blood vessels between basement membranes: (0, absent; 1, present). Character 50 in Nielsen et al. 1996.
2. Circulatory system a mixocoel, i.e. consisting of confluent haemal spaces and coelomic spaces: (0, absent; 1, present). Character 51 in Nielsen et al. 1996.
3. Heart with coelomic pericardium: (0, absent; 1, present). Character 52 in Nielsen et al. 1996.
4. Haemal system with axial complex: (0, absent; 1, present). Character 53 in Nielsen et al. 1996.
5. With protonephridia: (0, absent; 1, present). Character 54 in Nielsen et al. 1996.
6. With metanephridia, i.e. excretory ducts from coelomic compartments: (0, absent; 1, present). Character 55 in Nielsen et al. 1996.
7. Metanephridia with coelomic compartment restricted to a sacculus: (0, absent; 1, present). Character 56 in Nielsen et al. 1996.
8. Epithelia binding iodine and secreting iodothyrosine: (0, absent; 1, present). Character 57 in Nielsen et al. 1996.

**ADDITIONAL CHARACTERS FOR METAZOA**

1. Multicellularity: (0, absent; 1, present). Character 58 in Nielsen et al. 1996.
2. Spermatozoa: (0, absent; 1, present). Character 59 in Nielsen et al. 1996.
3. Collagen: (0, absent; 1, present). Character 60 in Nielsen et al. 1996.
4. Septate junctions: (0, absent; 1, present). Character 61 in Nielsen et al. 1996.
5. Choanocytes: (0, absent; 1, present).Ax 1995.
6. Extracellular digestion: (0, absent; 1, present).Ax 1995.
7. Ostia with porocytes: (0, absent; 1, present).Ax 1995.
8. Poriferan spicules: (0, absent; 1, present).The skeletal elements of sponges, not to be confused with octocorallian spicules.
9. Ciliary rosettes: (0, absent; 1, present).Character 13 in Ou et al. 2015.
10. Cydippid larvae: (0, absent; 1, present).Character 8 in Ou et al. 2015.
11. Embryonic development: (0, direct; 1, indirect). Character 9 in Ou et al. 2015.
12. Mitochondrial DNA: (0, circular; 1, linear).Character 2 in Marques & Collins 2004.
13. Cnidae: (0, absent; 1, present). Character 6 in Marques & Collins 2004.
14. Ctene rows: (0, absent; 1, present). Character 14 in Ou et al. 2015.
15. Aboral organ: (0, absent; 1, present). Character 25 in Ou et al. 2015.
16. Collocyte-bearing tentacles: (0, absent; 1, present). Character 45 in Ou et al. 2015.

**CHARACTERS FOR CNIDARIA**

- **Cnidome characters:**

1. Cnidocil: (0, mobile; 1, immobile).Character 1 in Marques & Collins 2004.
2. Stenoteles: (0, absent; 1, present).Character 7 in Marques & Collins 2004.
3. Euryteles: (0, absent; 1, present).Character 8 in Marques & Collins 2004.
4. Desmonemes: (0, absent; 1, present).Character 9 in Marques & Collins 2004.
5. Mastigophores: (0, absent; 1, present). Character 10 in Marques & Collins 2004.
6. Basitrichousisorhizas: (0, absent; 1, present). Character 11cin Marques & Collins 2004.
7. Apotrichousisorhizas: (0, absent; 1, present). Character 12 in Marques & Collins 2004.
8. Isorhizas (holotrichous or atrichous): (0, absent; 1, present). This characters combines the coding of the two different types of isorhizas to simplify differences in nomenclature and reportsbetween anthozoans and medusozoans.
9. Heterotrichousanisorhizas: (0, absent; 1, present). Character 14 in Marques & Collins 2004.
10. Birhopaloids: (0, absent; 1, present). Character 15 in Marques & Collins 2004.
11. Rhopalonemes: (0, absent; 1, present).Character 16 in Marques & Collins 2004.
12. Spirocyst: (0, absent; 1, present). Character 30 in Won et al. 2001.
13. Ptychocyst: (0, absent; 1, present). Character 31 in Won et al. 2001.

- **Tube characters:**

1. Periderm: (0, absent; 1, present). Derived from Mendoza-Becerril et al. 2016.
2. Cuticle layers: (0, one; 1, two). Derived from Mendoza-Becerril et al. 2016.
3. Chitin expression: (0, restricted to a basal area or to podocysts; 1, forming a tube; 2, forming a thin cuticle). Derived from Mendoza-Becerril et al. 2016.
4. Tube extension: (0, the tube encases all the polyp; 1, the tube does not cover the entire polyp). Derived from Mendoza-Becerril et al. 2016.
5. Periderm type: (0, corneous; 1, coriaceous). Derived from Mendoza-Becerril et al. 2016.

- **Characters of reproduction:**

1. Location of medusa formation: (0, lateral budding from an entocodon; 1, apical/oral; 2, directdevelopment without polyp stage). Character 20 in Marques & Collins 2004.
2. Type of apical medusa formation: (0, strobilation; 1, without transverse fission). Character 21 in Marques & Collins 2004.
3. Strobilation type: (0, polydisk; 1, monodisk). Character 22 in Marques & Collins 2004.
4. Propagation through lateral budding: (0, absent; 1, present). Naked ropaliophorans tend to produce secondary polyps as lateral branches which then separate and establish away from the progenitor. Also present in Hydra.
5. Oocyte development: (0, oocytes develop without accessory cells; 1, oocytes develop with accessory cells; 2, oocytes develop within follicles; 3, oocytes develop from uptake of somatic or other germ line cells). Character 23 in Marques & Collins 2004.
6. Spermatophore: (0, absent; 1, present). Character 24 in Marques & Collins 2004.

- **Siphonophore-only features:**

1. Nectosome: (0, absent; 1, present). Character 26 in Marques & Collins 2004.
2. Pneumatophore: (0, absent; 1, present). Character 27 in Marques & Collins 2004.

- **Characters of the planula:**

1. Planula larva in the life cycle: (0, present; 1, absent). Character 28 in Marques & Collins 2004.
2. Planula ciliation: (0, absent; 1, present). Character 29 in Marques & Collins 2004.
3. Number of endodermal cells of the planula: (0, varied number; 1, constant, *n*=16). Character 30 in Marques & Collins 2004.
4. Glandular cells in the planula: (0, absent; 1, present). Character 31 in Marques & Collins 2004.
5. Nervous cells in the planula: (0, absent; 1, present). Character 32 in Marques & Collins 2004.
6. Relationship between axes of planula and adult: (0, oral–aboral axis in the adult derived from the longitudinal axis of the planula; 1, oral–aboral axis in the adult derived from the transverse axis of the planula). Character 33 in Marques & Collins 2004.

- **Polyp characters:**

1. Polypoid phase: (0, absent; 1, present). Character 36 in Marques & Collins 2004. **We coded this character as being present in the fossil taxa because it is heavily implied by the developmental model we reconstruct for Pseudooides-Hexaconularia, and the other presumably closely related fossil taxa. This character is also established on the basis of a number of subordinate characters, that we consider, define what a polyp is in terms of their comparative anatomy: having an elongated radial body and a tapering end opposed to the presumed oral end of the animal.**
2. Polyp organization: (0, solitary; 1, colonial). Modified from character 1 in Daly et al. 2003. In the original there was another state that represented the presence of associations of clonal individuals. We decided to remove it because we think that state is reproductive, not anatomical.
3. Polymorphic polyps: (0, absent; 1, present). Character 37 in Marques & Collins 2004.
4. Number of tentacular whorls: (0, one; 1, two or more). Character 42 in Marques & Collins 2004.
5. Septa in polyp: (0, absent; 1, present). Modified from character 43 in Marques & Collins 2004. We decided to divide the original character to considerate the presence of septa regardless of the fine anatomy of these structures.
6. Mesoglea in polyp septa: (0, absent; 1, present). Also modified from character 43 in Marques & Collins 2004. In the original the absence of mesoglea in the septa was treated as a character state, we consider that the presence of septa itself is significant and introduce this character to differentiate between those who have the traditional scyphozoan and anthozoan type of septa from the likely derived state of cubozoans.
7. Pairing of mesentery: (0, paired; 1, coupled). Character 25 in Won et al. 2001.
8. Pair morphology: (0, members same size; 1, members differ in size). Character 20 in Daly et al. 2003.
9. Paired secondary cycle: (0, absent; 1, present). Character 21 in Daly et al. 2003.
10. Mesenterial fusion: (0, absent; 1, present). Character 23 in Daly et al. 2003.
11. Number of perfect mesenteries:(0, 8;1,10;2, morethan12).Character 18 in Won et al. 2001.
12. Types of mesentery: (0, only perfect mesenteries; 1, perfect mesenteries not divisible; 2, perfect mesenteries and imperfect mesenteries divisible). Character 19 in Won et al. 2001
13. Directive mesentery: (0, absent; 1, one pair; 2, two pairs). Character 22 in Won et al. 2001.
14. Mesentery formation: (0, only primary tentacles; 1, new septa arise in the exocoel to either side of the ventral directive; 2, from ventral; 3, between directive and transverse septa; 4, anywhere around the circumference). Character 23 in Won et al. 2001.
15. Stomodeum: (0, absent; 1, present). Character 45 in Marques & Collins 2004.
16. Structure of polyp tentacles: (0, hollow; 1, solid). Character 41 in Marques & Collins 2004.
17. Type of tentacles: (0, one type; 1, two types).Modified from character 15 in Won et al. 2001. In the original character the second state, “two types – long marginal and short labial” was intended to be specific of ceriantharians. We decided to open it to include any form of differentiation between different whorls of tentacles.
18. Clustered tentacles: (0, absent; 1, present). This character represents the presence of tentacles that are connected at their proximal parts.
19. Pinnate tentacles: (0, absent; 1, present).
20. Two-tentacle polyp stage: (0, absent; 1, present). The life cycle of staurozoans and cubozoans contains a polyp stage that only has two tentacles.
21. Siphonoglyph: (0, absent; 1, present).Modified from character 26 in Won et al. 2001. In the original publication this character discriminated between not having a siphonoglyph, having one and having either one or two. We decided to divide this character into two (chars. 136 and 137) because we consider that the presence or absence of siphonoglyphs overarchs the precise number of them.
22. Siphonoglyph count: (0, one; 1, more than one). Result from diving character 26 in Won et al. 2001.
23. Mesenteric filament: (0, consisting of 2 strips; 1, consisting of 3 strips; 2, consisting of just 1 strip). Character 20 in Won et al. 2001.
24. Ridges: (0, absent; 1, present). This character refers to a set of cuticular indentations that radiate into the gastrovascular regionof tube-bearing polyps of coronatids and all our fossil taxa.
25. Embryonic stage retained in the tube morphology: (0, absent; 1, present). This character refers to a particular character present in *Olivooides*, *Pseudooides* and *Quadrapyrgites*. Taxa that present it form their peridermal tubes directly from the embryo without a planula stage or getting fixed to a substrate.
26. Polyp symmetry: (0, radial; 1, bilateral; 2, birradial; 3, radial tetramerous; 4, pentamerous; **5, ten-fold symmetry**). Modified from character 18 in Marques & Collins 2004. Not applicable for taxa without a polyp stage.

- **Tentacles:**

1. Tentacles retractile: (0, no; 1, yes). Character 8 in Daly et al. 2003.
2. Tentacle/coelenteron relationship: (0,one tentacle per endocoel and per exocoel; 1, one tentacle per exocoel, multiple per endocoel).Character 9 in Daly et al. 2003.
3. Catch tentacles: (0, absent; 1, present). Character 12 in Daly et al. 2003.
4. Arrangement of tentacles: (0, scattered; 1, one cycle; 2, more than two cycles).Character 14 in Won et al. 2001.
5. Number of tentacles: (0, six; 1, eight; 2, more than 9). Character 17 in Won et al. 2001.
6. Acrospheres: (0, absent; 1, present). Character 13 in Daly et al. 2003.
7. Marginal spherules: (0, absent; 1,holotrichous). Character 14 in Daly et al. 2003.
8. Acontia: (0, absent; 1, present). Character 24 in Daly et al. 2003.
9. Gonads on mesenteries of 1st cycle: (0, absent; 1, present). Character 26 in Daly et al. 2003.
10. Gonads on mesenteries of 2nd and subsequent cycles: (0, absent; 1, present). Character 27 in Daly et al. 2003.
11. Mesogloeal sphincter: (0, absent; 1, present). Character 29 in Daly et al. 2003.
12. Ectodermal longitudinal muscle: (0, absent; 1, tentacles and oral disc only; 2, whole body). Character 30 in Daly et al. 2003.
13. Basilar musculature: (0, absent; 1, present). Character 31 in Daly et al. 2003.
14. Retractor muscle: (0, weak – not forming distinct muscle; 1, defined). Modified from character 32 in Daly et al. 2003. In the original character there were two states discriminating between different shapes of the muscle. We decided to simplify because the difference between the two states appeared to be faint and not widely described for taxa.
15. Parietal muscle: (0, absent; 1, present). Character 33 in Daly et al. 2003.
16. Mesogloeal lacunae: (0, absent; 1, present). Character 35 in Daly et al. 2003.
17. Acrorhagi: (0, absent; 1, present). Character 12 in Won et al. 2001.
18. Pedal disk: (0, absent; 1, present). Modified from character 13 in Won et al. 2001. In the original this character had three states: the pedal disk could either be absent, weak or strong. We consider the difference between it being strong or weak to be of minimal importance compared to the difference between having it or not.
19. Ciliated tract on mesenteric filament: (0, absent; 1, present). Character 21 in Won et al. 2001.

- **Post planula characters:**

1. Ephyrae: (0, absent; 1, present). Character 34 in Marques & Collins 2004.
2. Actinula: (0, absent; 1, present). Character 35 in Marques & Collins 2004.
3. Organisation of the nervous system: (0, in one or two nets; 1, with nerve rings). Character 46 in Marques & Collins 2004.
4. Canal system: (0, absent; 1, present). Character 47 in Marques & Collins 2004.
5. Gastrodermic musculature: (0, not organised in bunches; 1, organised in bunches of gastrodermic origin; 2, organised in bunches of ectodermic origin). Character 48 in Marques & Collins 2004.

- **Medusa-stage characters:**

1. Medusoid phase: (0, absent; 1, present). Character 49 in Marques & Collins 2004.
2. Pedalium of coronate type: (0, absent; 1, present). Character 50 in Marques & Collins 2004.
3. Rhopalia/rhopalioids: (0, absent; 1, present). Character 51 in Marques & Collins 2004.
4. Complex eyes in rhopalia: (0, absent; 1, present) Modified from character 52 in Marques & Collins 2004. We redefined this character from the original one that discerned three states of complexity into a binary character that is meant to separate the simpler rhopaliar organs from the clearly more complex organs present in Cubozoa.
5. Nerve ring(s): (0, absent; 1, one; 2, two). Character 53 in Marques & Collins 2004.
6. Gastric filaments: (0, absent; 1, present). Character 54 in Marques & Collins 2004.
7. Coronal muscle: (0, well developed; 1, marginal and tiny). Character 55 in Marques & Collins 2004.
8. Pedalium of the cubozoan type: (0, absent; 1, present). Character 56 in Marques & Collins 2004.
9. Velum: (0, absent; 1, present). Character 57 in Marques & Collins 2004.
10. Umbrellar margin: (0, smooth and continuous; 1, lobed). Character 58 in Marques & Collins 2004.
11. Tentacles: (0, absent; 1, present). Character 59 in Marques & Collins 2004.
12. Tentacular bulbs: (0, absent; 1, present). Character 60 in Marques & Collins 2004.
13. Statolith composition: (0, MgCaPO4; 1, CaSO4). Character 61 in Marques & Collins 2004.
14. Septa: (0, absent; 1, present). Character 62 in Marques & Collins 2004.
15. Septal shape: (0, straight; 1, Y-shaped). Character 63 in Marques & Collins 2004.
16. Radial canals: (0, absent; 1, present).Modified from character 64 in Marques & Collins 2004. We simplified the original three states into two, prioritising the difference between presence and absence.
17. Circular canal: (0, absent; 1, partially present; 2, fully present). Character 65 in Marques & Collins 2004.
18. Velarium: (0, absent; 1, present). Character 66 in Marques & Collins 2004.
19. Coronal furrow: (0, absent; 1, present). Character 67 in Marques & Collins 2004.
20. Gonadal location: (0, manubrium; 1, radial canals). Character 68 in Marques & Collins 2004.
21. Statocysts: (0, absent; 1, endodermic; 2, ectodermic). Character 69 in Marques & Collins 2004.
22. Perradial ‘mesenteries’: (0, absent, 1, present). Character 70 in Marques & Collins 2004.
23. Adult medusoid shape: (0, bell; 1, pyramidal; 2, cubic; 3, actinuloid). Character 71 in Marques & Collins 2004.
24. Shape of horizontal cross-section of the medusa:(0, circular; 1, four-part symmetry). Character 72 in Marques & Collins 2004.
25. Urticant rings: (0, absent; 1, present). Character 73 in Marques & Collins 2004.
26. Oral arms with suctorial mouths: (0, absent; 1, present). Character 74 in Marques & Collins 2004.
27. Tentacular insertion: (0, umbrellar margin; 1, away from the umbrellar margin). Character 75 in Marques & Collins 2004.
28. Manubrium: (0, absent; 1, present). Character 76 in Marques & Collins 2004.
29. Nervous system organisation: (0, GFNN absent; 1, GFNN present). Character 77 in Marques & Collins 2004.
30. Structure of medusa tentacles: (0, hollow; 1, solid). Character 78 in Marques & Collins 2004.
31. Tentacular morphology: (0, straight tentacles; 1, tentacles with an angular inflection). Character 79 in Marques & Collins 2004.
32. Peronia: (0, absent; 1, present). Character 80 in Marques & Collins 2004.
33. Ocelli: (0, absent; 1, present). Character 81 in Marques & Collins 2004.
34. Peripheral system: (0, absent; 1, present). Character 82 in Marques & Collins 2004.
35. Umbrellar furrow: (0, absent; 1, present). Character 83 in Marques & Collins 2004.
36. Development of the umbrella: (0, fully developed; 1, aboral cone). Character 84 in Marques & Collins 2004.
37. Number of tentacular whorls: (0, one whorl; 1, two whorls). Character 85 in Marques & Collins 2004.
38. Velar canals: (0, absent; 1, present). Character 86 in Marques & Collins 2004.
39. Frenulae: (0, absent; 1, present). Character 87 in Marques & Collins 2004.
40. Shape of tentacles of the medusa: (0, filiform; 1, capitate). Some cubozoans and staurozoans have clusters of tentacles.

- **Characters exclusive of anthozoans:**

1. Zooxanthellae: (0, absent; 1, present). Character 5 in Daly et al. 2003.
2. Ectodermal skeleton: (0, absent; 1, present). This character aims to differentiate taxa that produce their hard parts using their ectoderm fromothers that use their mesoglea to precipitate their skeletons.
3. Composition of the ectodermal skeleton: (0,proteinaceous; 1,calcitic). Within hexacorallians antipatharians have a proteinaceous skeleton whereas the rest of the hard part-bearing taxa have acalciticone.
4. Mesogleal skeleton: (0, absent; 1, present).Octocorallians, as opposed to hexacorallians, form their skeletons within their mesoglea.
5. Columella: (0, absent; 1, present). Character 37 in Daly et al. 2003.
6. Costae: (0, absent/weak; 1, present). Character 38 in Daly et al. 2003.

- **Characteres exclusive to Octocorallia:**

1. Peduncle or physa: (0, absent; 1, present).) Character 7 in Won et al. 2001.
2. Octocorallian calcareous spicules: (0, absent; 1, present). Modified from character 37 in Won et al. 2001. We redefined this character, specifying their octocorallian nature, to avoid confusion with the calcareous spicules of calcarean sponges.
3. Spicules in tentacle: (0, absent; 1, present). Character 39 in Won et al. 2001.
4. Gorgonin: (0, absent; 1, present). Character 40 in Won et al. 2001.

**Phylogenetic analysis:**

We assembled a morphological set to test the phylogenetic relationships of *Pseudooides* and other related taxa. The set includes representatives of three bilaterian phyla and anotherthree terminals to represent the Porifera, Placozoa and Ctenophora. Due to the fact that previous studies have concluded that some of these Early Cambrian embryos may be crown cnidarians (Dong et al. 2016), we included a wide array of cnidarians to test their affinities within the phylum.

To this end we put together a set of 47 taxa and 214 characters. The general characters for Metazoa are a combination of the morphological set of Nielsen et al. 1996, some of the general characters outlined in Ax 1995 and the synapomorphies of ctenophores in Ou et al. 2015. The set for Cnidaria is a combination of the largest set for medusozoans (Marques & Collins 2004) and two of the most comprehensive sets for Anthozoa (Won et al. 2001 and Daly et al. 2003). The new characters defined for tube morphologies in medusozoans come from Mendoza-Becerril et al. 2016 (Table.3). We followed the original coding for all non-modified characters. Simplified characters (three states into absence/presence) where coded as “present” for the different morphologies when they were not absent.

The phylogenetic analyses were run in MrBayes3.2 under the Mkv model (invgamma rates, variable coding). We ran four chains for 10 million generations twice. Both replicates reached convergence, and produced the same topologies albeit with slight differences in some clade credibilities. Summary statistics are based on the pooling of results from the two runs.
